# Microglia Polarization From M1 to M2 in Neurodegenerative Diseases

**DOI:** 10.3389/fnagi.2022.815347

**Published:** 2022-02-16

**Authors:** Shenrui Guo, Hui Wang, Yafu Yin

**Affiliations:** Department of Nuclear Medicine, Xinhua Hospital, Shanghai Jiao Tong University School of Medicine, Shanghai, China

**Keywords:** neurodegenerative diseases, neuroinflammation, Alzheimer’s disease, Parkinson’s disease, microglia polarization

## Abstract

Microglia-mediated neuroinflammation is a common feature of neurodegenerative diseases such as Alzheimer’s disease (AD), Parkinson’s disease (PD), amyotrophic lateral sclerosis (ALS), and multiple sclerosis (MS). Microglia can be categorized into two opposite types: classical (M1) or alternative (M2), though there’s a continuum of different intermediate phenotypes between M1 and M2, and microglia can transit from one phenotype to another. M1 microglia release inflammatory mediators and induce inflammation and neurotoxicity, while M2 microglia release anti-inflammatory mediators and induce anti-inflammatory and neuroprotectivity. Microglia-mediated neuroinflammation is considered as a double-edged sword, performing both harmful and helpful effects in neurodegenerative diseases. Previous studies showed that balancing microglia M1/M2 polarization had a promising therapeutic prospect in neurodegenerative diseases. We suggest that shifting microglia from M1 to M2 may be significant and we focus on the modulation of microglia polarization from M1 to M2, especially by important signal pathways, in neurodegenerative diseases.

## Introduction

Neurodegenerative diseases, a leading cause of morbidity and disability, are attracting increasing attention as the considerable impact on society (Stephenson et al., 2018). Microglia-mediated neuroinflammation is a common feature shared by various neurodegenerative diseases, including Alzheimer’s disease (AD), Parkinson’s disease (PD), amyotrophic lateral sclerosis (ALS), and multiple sclerosis (MS) (Song and Suk, 2017).

Neuroinflammatory includes microglial activation, and microglia could polarize into either M1 pro-inflammatory phenotype or M2 anti-inflammatory phenotype in response to different micro-environmental disturbances, which are called classical activation and alternative activation, respectively (Zhang B. et al., 2018). M1 microglia release inflammatory cytokines and chemokines, resulting in inflammation and neuronal death (Frank-Cannon et al., 2009). However, tissue maintenance and repair are associated with alternative activation of M2 microglia (Jha et al., 2016). M1 microglia induce inflammation and neurotoxicity, while M2 microglia induce anti-inflammatory and neuroprotection (Colonna and Butovsky, 2017), both of which are involved in the pathogenesis of neurodegenerative diseases, therefore microglia act as a double-edged sword in neurodegenerative diseases (Tang and Le, 2016). Fine modulation of microglia activation is essential for the normal function of microglia to keep brain homoeostasis and prevent neurodegenerative diseases (Song et al., 2016).

It’s impossible to repair or regenerate damaged neurons by current drugs nowadays once neurodegenerative diseases occur or even before the onset of diseases, but current drugs could alleviate disease-related symptoms by restricting the extent of neuroinflammation (Song and Suk, 2017). Fortunately, the balance between microglia M1/M2 polarization has a promising therapeutic prospect in the pathogenesis of neurodegenerative diseases (Song and Suk, 2017). However, previous studies have shown that several M1 inhibitive agents, such as cyclooxygenase inhibitors have no help in treating AD (Miguel-Álvarez et al., 2015). Becker et al. (2011) found the long-term use of non-steroidal anti-inflammatory drugs (NSAIDs), aspirin, or acetaminophen was also unhelpful in the risk of developing PD. Many other studies have shown that anti-inflammatory drugs such as aspirin, celecoxib and naproxen are not helpful in the prevention and treatment of neurodegenerative diseases (Cudkowicz et al., 2006; Lyketsos et al., 2007; Bentham et al., 2008; Jaturapatporn et al., 2012; Alzheimer’s Disease Anti-inflammatory Prevention Trial Research Group, 2013; Adapt-Fs Research Group, 2015; Poly et al., 2019; Ryan et al., 2020; Villarejo-Galende et al., 2020). Obviously, simple anti-inflammatory strategy may not be efficacious in clinical treatment of neurodegenerative diseases (Du et al., 2018).

As inhibiting M1 microglia alone is not enough, promoting M2 microglia activation simultaneously might also be required for treating neurodegenerative diseases (Yang et al., 2019). Promoting microglia polarization shift from M1 to M2 phenotype may be a more prospective strategy in the therapy of neurodegenerative diseases such as AD, PD, ALS, and MS (Wen et al., 2017; Zhang B. et al., 2018). Activated microglia is also a double-edged sword in other central nervous system diseases such as ischemic stroke, spinal cord injury and traumatic brain injury (Wang J. et al., 2018). There have been many studies about modulation of microglia polarization from M1 to M2 in these diseases (Zanier et al., 2014; Wang et al., 2015; Zhang et al., 2016; Wang J. et al., 2018; Jiang et al., 2020), which may provide many interesting ideas in neurodegenerative diseases.

In this review, we will focus on the role of microglia in neurodegenerative diseases such as AD, PD, ALS, and MS, and discuss the modulation of microglia polarization in these diseases to provide a promising therapeutic strategy.

## Functions and Phenotypes of Microglia

### Functions of Microglia

Microglia, resident macrophages in the brain, support the central nervous system (CNS) and are associated with the pathogenesis of many neurodegenerative diseases and other inflammatory diseases in the brain (Sarlus and Heneka, 2017). They originate from infiltrated yolk sac progenitor cells during early embryonic development, and are maintained independently by self-proliferation without circulating monocytes in normal conditions, while they are partially maintained by circulating monocytes under disease conditions (Ginhoux et al., 2010; Sarlus and Heneka, 2017).

Although microglia are brain resident immune cells, whose main functions are immune surveillance, immune defense and phagocytosis, they also provide trophic support to ensure tissue repair and keep homeostasis in the CNS (Sarlus and Heneka, 2017; Hansen et al., 2018). Besides, microglia are necessary during the development of CNS (Pierre et al., 2017). Microglia take part in neurogenesis, such as setting up neuronal circuitry and preserving the neuronal cell pool (Czeh et al., 2011; Pierre et al., 2017). Microglia take part in synaptic pruning through removing unnecessary neurons and synapses, and supporting survival and differentiation of neurons, thus playing an important role in setting up normal neuronal connectivity (Eyo and Dailey, 2013). Microglia take part in myelination and the establishment of normal vascularization of the brain and the retina (Pierre et al., 2017). Microglia are also associated with higher cognitive functions such as learning and memory by promoting the formation of learning-related synapses through brain-derived neurotrophic factor (BDNF) signal transduction (Parkhurst et al., 2013).

There are two states of microglia, resting and activated (Cherry et al., 2014). Microglia are at rest in the healthy brain, with rod-shaped soma as well as ramified and tiny processes (Carta and Pisanu, 2013). When detecting changes in brain homeostasis, microglia undergo morphological and functional changes, which is called microglia activation (Choi et al., 2012). Once activated, microglia rapidly retract and shorten their processes, and enlarge their soma (Carta and Pisanu, 2013).

### Phenotypes of Microglia

As influenced by the environment, microglia assume a diversity of phenotypes and have the ability to shift phenotypes to keep tissue homeostasis (Orihuela et al., 2016). Microglial activation in the CNS is heterogeneous, which can be categorized into two opposite types: classical (M1) or alternative (M2), though it seems there is a continuum of phenotypes between M1 and M2 (Sica and Mantovani, 2012; Tang and Le, 2016).

M1 activation is known as classical activation, which is typically induced by interferon-γ (IFN-γ) and lipopolysaccharide (LPS) (Colonna and Butovsky, 2017). M1 microglia produce inflammatory cytokines and chemokines, such as tumor necrosis factor alpha (TNF-α), interleukin (IL)-6, IL-1β, IL-12, and CC chemokine ligand (CCL) 2 (Colonna and Butovsky, 2017). They express nicotinamide adenine dinucleotide phosphate (NADPH) oxidase [which produces superoxide and reactive oxygen species (ROS)] and inducible nitric oxide synthase [iNOS, which produce nitric oxide (NO)], major histocompatibility complex-II (MHC-II), integrins (CD11b, CD11c), costimulatory molecules (CD36, CD45, CD47), and Fc receptors (Colonna and Butovsky, 2017), contributing to neurological damage (Nguyen et al., 2017).

M2 activation is known as alternative activation, which is induced by anti-inflammatory cytokines such as IL-4 and IL-13 (Colonna and Butovsky, 2017). M2 microglia produce anti-inflammatory cytokines [IL-10, transforming growth factor (TGF)-β)], growth factors [insulin-like growth factor-1 (IGF-1), fibroblast growth factor (FGF), colony stimulating factor (CSF)−1] and neurotrophic growth factors [nerve-derived growth factor (NGF), BDNF, neurotrophins and glial cell-derived neurotrophic factor (GDNF)] (Colonna and Butovsky, 2017). They also release pro-survival factor progranulin and induces mannose receptor (CD206), found in inflammatory zone 1 (FIZZ1), chitinase-3-like-3 (Chil3, Ym1 in rodents), arginase 1 (Arg1) (Colonna and Butovsky, 2017). M2 microglia promote phagocytosis of cell debris and misfolded proteins, promote extracellular matrix reconstruction and tissue repair, and support neuron survival by neurotrophic factors (Tang and Le, 2016).

On the whole, M1 microglia induce inflammation and neurotoxicity, while M2 microglia induce anti-inflammatory and healing (Colonna and Butovsky, 2017). However, transcriptome studies have shown that microglia activation is varied, meaning that M1 and M2 represent a spectrum of activation patterns rather than separate cell subtypes (Correale, 2014), and there’s a continuum of different intermediate phenotypes in microglia, thus the M1/M2 paradigm is inadequate to accurately describe microglia activation *in vivo* (Colonna and Butovsky, 2017). Microglia can transit from one phenotype to another according to different environments in the CNS (Correale, 2014), ensuring that microglia play a protective role by switching their phenotype (Du et al., 2018).

Since marker based analyzing methods have limits to define the cell types and states of microglia, Keren-Shaul et al. (2017) combined massively parallel single-cell RNA-seq (MARS-seq), single-molecule fluorescent *in situ* hybridization (smFISH), chromatin immunoprecipitation (iChiP) and etc. to comprehensively characterize microglia in AD. They described a novel sub-population of microglia associated with neurodegenerative diseases: disease-associated microglia (DAM) (Keren-Shaul et al., 2017). As AD progresses, microglia could transit from homeostatic microglia to DAM, which are localized around the amyloid-β (Aβ) plaques (Keren-Shaul et al., 2017). DAM activation is a two-step process: the first step leads homeostatic microglia to an intermediate state in a Trem2-independent mechanism, involving decreased expression of homeostatic microglia checkpoint genes such as *Cx3cr1* and purinergic receptors *P2ry12/P2ry13*, and up-regulation of genes such as *B2m*, *ApoE*, and the Trem2 adaptor *Tyrobp*; and the following second step is Trem2-dependent, involving up-regulation of phagocytic and lipid metabolism genes such as *Lpl*, *Cst7*, and *CD9* (Keren-Shaul et al., 2017). Similar DAM subpopulation was found in ALS models as well (Keren-Shaul et al., 2017). DAM subpopulation is more specific than the binary classification of microglia, and DAM are likely to be a potential therapeutic direction for neurodegenerative diseases as their protective effects.

Although the binary concept of microglial M1/M2 classification has recently been debated, classifying microglia function as either neurotoxic (M1) or neuroprotective (M2) is useful for explaining the pathobiology of inflammatory and degenerative CNS disorders (Song and Suk, 2017). In this review, we will adopt the binary classification of microglia to discuss microglia polarization from M1 to M2 phenotype in neurodegenerative diseases.

## Microglia Polarization From M1 to M2

Many modulators are involved in regulating microglia polarization, which can be divided into several categories, including transcription factors, receptors, cytokines, ion channels, bioactive compounds, drugs, and so on. However, the certain mechanism in microglia polarization is still not distinct, while potential exploitation of these modulators may be favorable to shift microglia from M1 to M2 phenotype. Indeed, there have been plenty of studies about this issue *in vitro* or in wild type mice and other diseases such as ischemic stroke, spinal cord injury, and traumatic brain injury (Zanier et al., 2014; Wang et al., 2015; Zhang et al., 2016; Wang J. et al., 2018; Jiang et al., 2020). Similar studies in the specific disease environment of neurodegenerative diseases are rare.

### Signal Pathways

**FIGURE 1:**
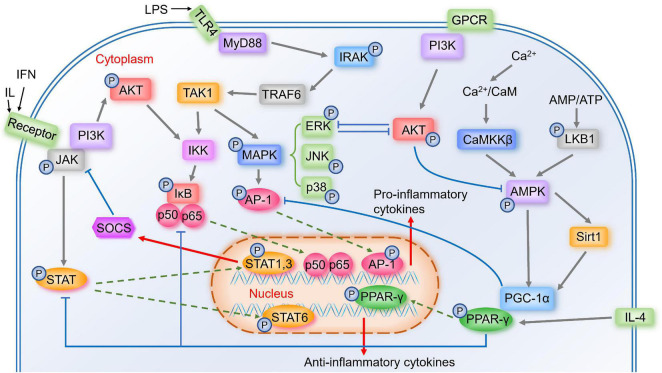
Important signal pathways in the modulation of microglia polarization. Activated TLR4 associates with MyD88 to induce the autophosphorylation of IRAK, which interacts with TRAF6. TRAF6 then activates the TAK1, activating two major pro-inflammatory pathways, IKK/IκB/NF-κB and MAPK (including ERK, JNK, p38)/AP-1 signaling pathways, thus the pro-inflammatory genes transcribe. Activated JAK induces the phosphorylation of STAT, and STAT1&STAT3 induce expression of pro-inflammatory mediators and negative feedback regulator SOCS. While STAT6 is involved in microglial M2 polarization. Calcium/CaMKKβ/AMPK signaling pathway is activated by Ca^2+^/CaM or LKB1, the latter is activated by increased AMP/ATP ratio. AMPK activates Sirt1, leading to the activation of PGC-1α, while AMPK also phosphorylates PGC-1α directly. PGC-1α interacts with PPAR-γ, which is involved in IL-4-induced M2 polarization. PPAR-γ antagonizes AP-1, NF-κB, and STAT1. GPCR and phosphorylated JAK could activate PI3K/AKT pathway. AKT prevents AMPK activation, and ERK and AKT inhibit each other.

Several important signal pathways are involved in microglia polarization, and blocking the components of these signal pathways is likely to influence the phenotype of microglia. First, we will discuss some important signal pathways in modulating microglia polarization, as many other modulators are associated with these signal pathways (Figure 1).

#### Toll-Like Receptor Signal Pathways

TLR including TLR2 and TLR4 belong to transmembrane pattern-recognition receptor family, which are highly expressed in immune cells like microglia (Cui et al., 2020). One ligand of TLR4 is lipopolysaccharide (LPS) (Lehnardt et al., 2003), a major component of the outer membrane of Gram-negative bacteria (Ciesielska et al., 2021), which is a strong factor in regulating inflammatory mediators and M1 microglia polarization (Yang et al., 2019). LPS binds to LPS-binding protein and the soluble or glycosylphosphatidylinositol-anchored CD14, and further this complex binds to TLR4, starting a pro-inflammatory signaling cascade (Lehnardt et al., 2003). Once activated, TLR4 associates with the adaptor protein myeloid differentiation factor 88 (MyD88) and induces the autophosphorylation of interleukin-1 receptor-associated kinase (IRAK) (Subhramanyam et al., 2019). Phosphorylated IRAK4 and IRAK1 then dissociate from MyD88 and interact with tumor necrosis factor receptor associated factor-6 (TRAF6) (Subhramanyam et al., 2019). Later, TRAF6 activates the transforming growth factor-β-activated kinase-1 (TAK1) complex, and then activates two major pro-inflammatory pathways, nuclear factor kappa-B (NF-κB) and mitogen-activated protein kinases (MAPK) signaling pathways, transcribing the pro-inflammatory genes (Kawai and Akira, 2007; Subhramanyam et al., 2019). TLR2 also plays an important role in the innate immune system by MyD88 (Cui et al., 2020). Ma et al. (2020) found that autophagy was associated with TLR2 signaling, and TLR2-mediated autophagy could regulate microglial M1/M2 phenotype switching.

#### Nuclear Factor Kappa-B Signal Pathways

NF-κB is a key transcription factor related to M1 microglial activation, and Zhang et al. (2013) showed that inhibiting NF-κB p65/p50 subunits suppressed the transcription of inflammatory genes and drove microglia to M2 phenotype. Many modulators could shift microglia from M1 to M2 by inhibiting NF-κB (Kawai and Akira, 2007), and many other signal pathways could affect NF-κB pathway, suggesting that NF-κB may play a crucial role in microglia polarization. Without stimulation, NF-κB is inactive in the cytoplasm by interacting with inhibitors of NF-κB (IκB) proteins (Kawai and Akira, 2007). Activated TAK1 complex activates the IκB kinase (IKK) complex, which catalyzes the phosphorylation of IκB proteins (Kawai and Akira, 2007). Then phosphorylated IκBs are polyubiquitinated and degraded, allowing NF-κB p50/p65 to translocate into the nucleus (Kawai and Akira, 2007), where they bind to target promoters and then the pro-inflammatory genes transcribe (Mulero et al., 2019).

#### Mitogen-Activated Protein Kinases Signal Pathway

Activated complex TAK1 also activates the MAPK pathway, which results in the phosphorylation and activation of transcription factor activator protein-1 (AP-1) (Kawai and Akira, 2007). MAPK is comprised of the p42/44 extracellular signal-regulated kinase (ERK) MAPKs, c-Jun NH2-terminal kinases (JNKs), and p38 MAPK (Ji et al., 2010). MAPK signaling exerts anti-inflammatory effects as well as promotes microglia polarization to M2 (Zhang B. et al., 2018).

#### Janus Kinase/Signal Transducer and Activator of Transcription Signal Pathway

The Janus kinase/signal transducer and activator of transcription (JAK/STAT) signaling plays an important role in immune and inflammatory responses (Ruganzu et al., 2021). STAT1 and STAT3 mediate M1 microglia polarization, inducing pro-inflammatory cytokines and chemokines (Lan et al., 2017). While STAT6 is activated by IL-4 (Lan et al., 2017), which may promote microglia M2 polarization. Activated JAK induces the phosphorylation of STAT family proteins (Cianciulli et al., 2017). Phosphorylated STAT then translocates to the nucleus and starts the transcription of targeted genes (Durham et al., 2019), including suppressor of cytokine signaling (SOCS) (Xin et al., 2020). As key negative feedback regulators, SOCS family proteins could inhibit the phosphorylation of JAK (Durham et al., 2019), and several studies have shown that unregulated SOCS1 and SOCS3 could attenuate inflammatory responses by switching microglia from M1 to M2 (Ruganzu et al., 2021).

#### AMP-Activated Protein Kinase Signal Pathway

During inflammation, intracellular Calcium (Ca^2+^) influx increases (Dalal et al., 2020). Ca^2+^ binding to calmodulin (CaM) triggers the activation of a highly conserved Ca^2+^/CaM kinase cascade including Calcium/calmodulin-dependent protein kinase kinase-β (CaMKKβ) (Marcelo et al., 2016). Activated CaMKKβ phosphorylates AMP-activated protein kinase (AMPK) (Marcelo et al., 2016). AMPK is a key energy sensor in many tissues (Liu and Chern, 2015) and plays a role in microglia polarization from M1 to M2 (Wang Y. et al., 2018). Another upstream kinase that phosphorylates and activates AMPK is liver kinase B1 (LKB1), which is activated by the increased AMP/ATP ratio (Dengler, 2020).

AMPK can activate peroxisome proliferator-activated receptor γ coactivator 1α (PGC-1α) directly, and it also increases NAD^+^ amounts to activate Sirtuin 1 (Sirt1), leading to the deacetylation and activation of PGC-1α (Fernandez-Marcos and Auwerx, 2011). PGC-1α was initially identified through interacting with peroxisome proliferator-activated receptor gamma (PPAR-γ), leading to an increase in PPAR-γ transcriptional activity (Zhang Y. et al., 2020).

#### PI3K/AKT Signaling Pathway

The Ser and Thr kinase AKT, also known as protein kinase B (PKB), is initiated by the stimulation of G-protein-coupled receptors (GPCR) or receptor tyrosine kinases (RTK), leading to the activation of phosphoinositide 3-kinase (PI3K), thus causes the phosphorylation of T308 and S473 residues of AKT (Manning and Toker, 2017). Several researches showed that low levels of PI3K/AKT signaling induced M1 phenotype, while enhanced PI3K/AKT signaling induced M2 phenotype (Linton et al., 2019). There are several cross talks between the PI3K/AKT pathway and other major signaling pathways. For example, NF-κB is a downstream component of the PI3K/AKT pathway (Yu et al., 2017), and phosphorylated JAK is essential in the activation of the PI3K/AKT pathway (Gao et al., 2018). AKT indirectly prevents AMPK activation by stimulating ATP production or directly phosphorylating AMPK, which hinders LKB1 to phosphorylate AMPK (Manning and Toker, 2017). ERK activation suppresses PI3K/AKT pathway, and AKT inhibits ERK pathway as well (Manning and Toker, 2017).

#### Rho/ROCK Signaling Pathway

Rho-associated protein kinase (ROCK), a serine/threonine kinase, is a potential regulator of microglia phenotype (Song and Suk, 2017). Rho, one of the most important members of the Rho GTPases, which belongs to Ras superfamily (Xu et al., 2020), is an upstream activator of ROCK (Koch et al., 2018). Rho/ROCK signaling pathway plays an important role in inflammation (Deng et al., 2019; Xu et al., 2020). ROCK inhibitor Fasudil reduces pro-inflammatory factors NO, IL-1β, IL-6, and TNFα and increases anti-inflammatory factor IL-10 in microglia, suggesting that low ROCK2 activity shifts microglia from M1 to M2 (Koch et al., 2018). Chen et al. (2017) showed that Fasudil also led to a M2 phenotype by NF-κB p65.

#### Notch Signaling Pathway

Notch signaling pathway, a highly conserved intracellular signaling route, occurs in a cell-cell contact-dependent manner (Nowell and Radtke, 2017). There are four Notch receptors (NOTCH 1–4) and five ligands of the Delta-Serrate-Lag (DSL) family [jagged 1 (JAG1), JAG2, delta-like 1 (DLL1), DLL3, and DLL4] (Nowell and Radtke, 2017). The transmembrane receptor of Notch is generated in the endoplasmic reticulum and transported to the plasma membrane, whose interaction with a transmembrane ligand on a neighboring cell results in separation of receptor subunit and proteolytic cleavage of the transmembrane subunit to release a Notch intracellular domain (NICD) (Majumder et al., 2021). The NICD translocates to the nucleus, where it acts as a transcriptional co-activator and promotes the expression of target genes (Majumder et al., 2021), thus regulating a variety of cellular processes, such as proliferation, stem cell maintenance, differentiation, death (Majumder et al., 2021), as well as microglia polarization (Wu et al., 2018).

#### Transcription Factor

NF-κB, AP-1, and STAT are important transcription factors related to M1 microglia polarization, which transcribe pro-inflammatory genes. Researches on these transcription factors suggest that inhibiting these transcription factors probably results in microglia polarization from M1 to M2.

PPAR-γ, a nuclear receptor superfamily of ligand-inducible transcription factor, is highly expressed in microglia (Carta and Pisanu, 2013). IL-4 induces M2 microglia through PPAR-γ signaling pathway (Jiang et al., 2020). PPAR-γ modulates the production of both pro- and anti-inflammatory cytokines in microglia, and up-regulate the M2 marker Arg-1, acting as a crucial regulator in M2 polarization (Ahmadian et al., 2013; Pisanu et al., 2014; Xu et al., 2015; Song et al., 2016; Song and Suk, 2017). PPAR-γ exerts anti-inflammation effect by antagonizing inflammatory transcription factors including AP-1, NF-κB, and STAT1 (Jacobi et al., 2012). For example, PPAR-γ activation blocks NF-κB signaling pathway by inhibiting nuclear translocation of p65 subunit or competing with NF-κB co-activators (Yang et al., 2019). Ji et al. (2018) found that antagonizing PPAR-γ promoted M1 to M2 through improving autophagy by promoting the LKB1/AMPK signaling pathway.

### Receptors

Several receptors could promote M2 polarization, such as triggering receptor expressed on myeloid cells 2 (TREM2), a type I transmembrane receptor of microglia (Sekiyama et al., 2012). Sekiyama et al. (2012) showed that knockout of TREM2 in BV2 microglia inhibited M2 polarization and led to an increase of M1 microglia inflammatory responses, whereas overexpression of TREM2 promoted M2 polarization and reduced microglia-mediated inflammation. Zhang et al. (2017) found that acetylcholine (Ach) transformed M1 microglia to M2 through promoting α7 subtype of the nicotinic acetylcholine receptor (α7 nAChR) by JAK2/STAT3 pathway. NADPH-oxidase activation promotes the M1 phenotype and inhibits the M2 phenotype (Labandeira-Garcia et al., 2017b), which can be activated by angiotensin II via angiotensin II type I (AT1) receptor (Labandeira-Garcia et al., 2017b). Thus, blockade of brain AT1 receptor is neuroprotective (Saavedra et al., 2011), probably related to M2 phenotype. Purinergic receptor P2 × 7 is also related to microglia polarization (Apolloni et al., 2014).

### Cytokines

Cytokines may shift microglia from M1 toward M2 as well, such as anti-inflammatory cytokines IL-4 (Tang et al., 2019), IL-13 (Colonna and Butovsky, 2017), IL-10, TGF-β (Tang and Le, 2016), and GDNF (Zhong et al., 2020), insulin-like growth factor-1 (IGF-1) (Labandeira-Garcia et al., 2017a), milk fat globule epidermal growth factor 8 (MFG-E8) (Shi et al., 2017), and IL-33 (Liu et al., 2010; Fu et al., 2016). Zhong et al. (2020) found that adipose-derived stem cells (ADSCs) could produce GDNF in an inflammatory microenvironment, which exerted neuroprotective effects in neurological diseases, thus modulating microglia polarization to M2 phenotype through promoting PI3K/AKT signaling pathway.

### Ion Channels

The expression of ion channels changes in response to voltage in the microenvironment, which induces intracellular signal transduction (Jiang et al., 2020). Recent research showed that K_*v*_1.3 and Kir6.1 were closely associated with microglia polarization (Du et al., 2018; Maezawa et al., 2018).

### Bioactive Compounds

Many bioactive compounds exert anti-inflammatory and neuroprotective effects, most of which could change the phenotype of microglia by signal pathways mentioned above. Astaxanthin and anisalcohol could shift microglia from M1 to M2 by inhibiting NF-κB and JNK signaling (Wen et al., 2017; Xiang et al., 2018). Naringenin could shift microglia from M1 to M2 by inhibiting MAPK signaling activation, especially JNK signaling (Zhang B. et al., 2018). Sirt1 agonist resveratrol could shift microglia from M1 to M2 by increasing PGC-1α expression, inhibiting NF-κB pathway, and activating STAT6 and STAT3 pathways (Yang et al., 2017). Platycodigenin could shift microglia from M1 to M2 by inhibiting p38, and activating PPAR-γ, thus blocking NF-κB pathway (Yang et al., 2019). Hydroxytyrosol could shift microglia from M1 to M2 by inhibiting NF-κB P65 and ERK signaling pathway (Zhang et al., 2020a). Curcumin could shift microglia from M1 to M2 by suppressing TLR4/NF-κB pathways, and down-regulating TREM2 or stimulating CaMKKβ-dependent AMPK signaling pathway (Zhang et al., 2019; Qiao et al., 2020). While Porro et al. (2019) also found that curcumin could elicit anti-inflammatory responses via JAK/STAT/SOCS signaling pathway. Isovitexin and Betulinic acid could shift microglia from M1 to M2 by CaMKKβ/AMPK signaling pathway (Li et al., 2018; Liu et al., 2019b). Rosmarinic acid could shift microglia from M1 to M2 via the phosphoinositide-dependent protein kinase 1 (PDPK1)/Akt/hypoxia-inducible factor (HIF) pathway, which are involved in mitochondrial respiration and coordinating the metabolic and functional reprogramming of M1/M2 microglia polarization (Wei et al., 2021).

### Drugs

Similarly, drugs are likely to play an important role in microglia polarization by these signal pathways as well. Qie et al. (2020) found that candesartan, an AT1 receptor blocker (ARB), modulated the neuroinflammatory response, reversed neurotoxic effect and shifted microglia from M1 to M2 at least partially by inhibiting TLR4/NF-κB signaling pathway. Xu et al. (2015) also found another ARB telmisartan promoted M2 polarization and reduced M1 polarization through PPAR-γ and CaMKKβ/AMPK pathway. Dexmedetomidine, an alpha2 adrenoceptor agonist, promotes M2 polarization by inhibiting ERK1/2 signaling (Qiu et al., 2020). Wu et al. (2018) suggested that simvastatin, a hydroxymethylglutaryl–coenzyme A (HMG-CoA) reductase inhibitor, regulated microglia polarization from M1 to M2 by Notch signaling pathway. Nicotine, a neuronal nicotinic acetylcholine receptor (nAChR) agonist, promotes M2 microglia polarization via activation of STAT3 pathway (Wu et al., 2020).

### Others

While some other modulators exert phenotypic transformation effect in their specific ways. Inhibiting NADPH oxidase (Nox), which is responsible for producing ROS, drives microglia phenotype from M1 to M2 (Babior, 1999; Choi et al., 2012). CDGSH iron-sulfur domain 2(CISD2), a longevity gene, exerts anti-inflammatory effects by inhibiting NF-κB signaling and preserving M2 microglia (Lin, 2020). Toscano et al. (2020) showed that postprandial triglyceride-rich lipoproteins (TRLs) could modulate microglia polarization in a fatty-acid-dependent manner: postprandial TRL-monounsaturated fatty acids promote M2 microglia polarization, whereas postprandial TRL-saturated fatty acids improve M1 microglia polarization. Melatonin can ameliorate neurobehavioral disturbances and alleviate axonal hypomyelination in the periventricular white matter of LPS-injected postnatal rats, and reduces neuroinflammation through shifting M1 microglia toward M2 via melatonin receptor 1/JAK2/STAT3/telomerase pathway (Zhou et al., 2021).

## Microglia Polarization From M1 to M2 in Neurodegenerative Diseases

Neurodegenerative diseases including AD, PD, ALS, and MS are characterized by degeneration of neurons in specific regions of the CNS, and share common pathophysiological mechanisms such as neuroinflammation and deposition of abnormal proteins (Song and Suk, 2017). The role of microglia and the mechanism of microglia polarization in different neurodegenerative diseases are similar. Microglia-mediated neuroinflammation is a common feature of neurodegenerative diseases, and several inflammatory mediators released by M1 microglia are involved in the pathogenesis of neurodegeneration and myelin injury in these diseases (Song and Suk, 2017). However, tissue maintenance and repair need M2 microglia activation (Jha et al., 2016). Microglia-mediated neuroinflammation is regarded as a double-edged sword in neurodegenerative diseases, carrying out both harmful and helpful effects on neurons and the nearby environment (Tang and Le, 2016).

### Microglia Polarization From M1 to M2 in Alzheimer’s Disease

AD is the most common dementia, associated with progressive memory loss and cognitive decline, such as damaged locomotor ability, reasoning, and judgment ability (Fu et al., 2016; Sarlus and Heneka, 2017). AD is characterized by brain atrophy, amyloid plaques [formed by accumulation of extracellular amyloid-β (Aβ) peptides, which derived from the cleavage of amyloid precursor protein (APP) with the help of presenilin-1 (PS1)], intracellular neurofibrillary tangles (formed by the deposition of hyperphosphorylated tau), with loss of neurons and synapses, and dystrophic neuritis (Tang and Le, 2016; Sarlus and Heneka, 2017; Hansen et al., 2018).

As resident macrophages in the brain, microglia can response to harmful stimuli like misfolded Aβ proteins (Sarlus and Heneka, 2017). Microglia play a protective role in the brain, known as M2 microglia, which are mainly responsible for uptaking and removing insoluble fibrillar Aβ deposits (Heneka, 2017). Microglia can degrade Aβ by secreting enzymes such as insulin−degrading enzyme (Heneka, 2017), thus preventing AD (Hansen et al., 2018). Microglia play a critical role in compacting and corralling amyloid deposits, encapsulating them as a protective barrier, thus limiting accumulated potentially neurotoxic Aβ aggregates as well as shielding neurons from local toxicity (Condello et al., 2015). Compared with other cell types in the brain, many AD-related genetic variants are selectively or preferentially expressed in microglia, such as *TREM2*, *APOE*, *CD33*, *INPP5D*, *MS4A6A*, and *PLCG2* (Srinivasan et al., 2016; Sarlus and Heneka, 2017; Hansen et al., 2018).

Proper microglia function is protective in AD, while there’s great evidence that unbridled microglia activation and altered microglia responses to Aβ can be harmful to neurons (Hansen et al., 2018). Once overactivated, microglia show increased proliferation, induce chemotaxis and increase inflammatory M1 markers (Heneka, 2017; Sarlus and Heneka, 2017). Microglia secrete neurotoxic cytokines which injure neurons directly or by activating neurotoxic astrocytes (Hansen et al., 2018). Microglia also engulf synapses with the help of complement, and provoke the spread of tau pathology (Hansen et al., 2018).

Microglial activation is dynamic, and they undergo continuous transformation among their phenotypes (Fan et al., 2017). Fan et al. (2017) speculated there might be two peaks of microglial activation in AD: an early anti-inflammatory peak in the preclinical stage and a later pro-inflammatory peak in the clinical stage as disease progresses with the failure in Aβ clearance, conforming to the dual role of microglia in AD pathogenesis.

### Microglia Polarization From M1 to M2 in Parkinson’s Disease

PD is the second-most prevalent neurodegenerative disease after AD, which is characterized by a progressive degeneration of dopamine (DA) neurons in the substantia nigra compacta (SNc) of the midbrain, accumulation of abnormal proteins like α-synuclein in Lewy bodies and excessive reactive microglia proliferation (Tang et al., 2014; Song and Suk, 2017; Zhang Y. et al., 2018). Microglia-mediated neuroinflammation also underlies the pathogenesis of PD, which could be a double-edged sword that is neuroprotective in the early stage but becomes harmful as PD progresses (Sekiyama et al., 2012; Zhang Y. et al., 2018). Microglia are activated by α-synuclein, pathogens or environmental toxins in the early stage of PD, remaining relatively static, and are irrelevant with clinical severity (Tang and Le, 2016). Microglia release anti-inflammatory cytokines to reduce inflammatory responses and enhance the expression of genes related to tissue recovery and repair (Du et al., 2018). Although microglial activation is essential and critical for immune defense and neuron survival (Tang et al., 2014), continued microglial activation is generally harmful (Song and Suk, 2017). Aggregated α-synuclein could directly induce microglia toward M1 phenotype (Tang and Le, 2016), which aggravates motor deficits and expands extensive neuronal damage to neighboring neurons in PD (Song and Suk, 2017; Du et al., 2018).

### Microglia Polarization From M1 to M2 in Amyotrophic Lateral Sclerosis

Amyotrophic lateral sclerosis (ALS), a late-onset neurodegenerative disease, is characterized by progressive degeneration of motor neurons in the spinal cord, brain stem and primary motor cortex, eventually leading to paralysis and death (Huang et al., 2012; Geloso et al., 2017). Mutations in several genes contribute to the development of ALS, such as expansion of a hexanucleotide repeat (GGGGCC) in the C9orf72 gene, the Cu^2+^/Zn^2+^ superoxide dismutase (mSOD1), and transactive response (TAR) DNA binding protein 43 kDa (TDP-43) (Boillée et al., 2006; Huang et al., 2012; Tang and Le, 2016; McCauley et al., 2020). Microglia are easily induced to M1 phenotypes with aggregated mutant forms of mSOD1 and TDP-43 proteins (Zhao et al., 2010, 2015; Tang and Le, 2016). Similarly, microglia have both neuroprotective and neurotoxic functions in ALS (Tang and Le, 2016). Several studies have proved that microglia show M2 phenotype and protect motoneurons at the onset of the disease, while end-stage microglia shift to M1 phenotype and aggravate the injury of motor neurons in ALS mice (Liao et al., 2012; Gravel et al., 2016). In mSOD1^*G*93*A*^ mouse model, microglia appear to switch from M2 phenotype noted at the beginning of pathology to M1 phenotype as disease progresses (Tang and Le, 2016). Similarly, Liao et al. (2012) found that microglia expressed higher levels of M2 markers and lower levels of M1 markers at an early stage compared with the late stage in ALS mice.

### Microglia Polarization From M1 to M2 in Multiple Sclerosis

MS is the most common multifocal inflammatory demyelination occurring in the white matter of the CNS, and it’s also a T-cell mediated autoimmune disease (Miron et al., 2013), which is characterized by monocyte infiltration, microglial activation, demyelination, and injury of axon and neuron (Chen et al., 2014; Song and Suk, 2017). The spinal cord barrier (BSCB) is a protective barrier in the CNS similar to BBB, whose disruption is a primary and persistent pathological feature of MS (Zhang et al., 2020b). Experimental autoimmune encephalomyelitis (EAE) is a widely used model of MS. Neuroinflammation plays an important role in neurodegeneration in MS (Correale, 2014). The development of EAE is mediated by the interplay between reactive T cells and microglia (Martinez-Pasamar et al., 2013). Activated microglia acts as first line defenders against infections or inflammation in EAE (Chastain et al., 2011). Miron et al. (2013) showed that microglia are immediately activated to M1 phenotype during the early phase, and shift to M2 phenotype during the later phase. Microglia release neurotoxic and pro-inflammatory cytokines and chemokines like macrophage inflammatory protein (MIP) and CCL (Miron et al., 2013), which damage oligodendrocytes and neurons, contributing to demyelination and neurodegeneration in early lesions of MS/EAE (Chastain et al., 2011; Correale, 2014). Microglia could recruit other immune cells into the CNS to enlarge the inflammation response (Jiang et al., 2014). Microglia also act as antigen presenting cells presenting myelin antigen to autoreactive T cells to perpetuate the self-destructive environment during chronic inflammation (Chastain et al., 2011). Miron et al. (2013) found a switch from M1 to M2 in microglia at the initiation of remyelination. M2 microglia can release neurotrophic molecules and anti-inflammatory cytokines during recovery (Song and Suk, 2017), contributing to oligodendrocyte differentiation and remyelination (Miron et al., 2013). In that, microglia play both helpful and harmful roles in the demyelination and recovery stages, respectively (Song and Suk, 2017).

## Receptors Involved in Microglia Polarization in Neurodegenerative Diseases

Heterozygous rare variants in *TREM2* (rs75932628, p.R47H) are associated with a significantly increased risk of AD (Guerreiro et al., 2013). TREM2 is an important regulator of M2 microglia polarization in AD (Sekiyama et al., 2012). Ruganzu et al. (2021) showed that overexpressed TREM2 rescued cognitive deficits, decreased Aβ plaques deposition, reduced synaptic and neuronal loss, ameliorated neuroinflammation, promoted M2 polarization as well as reduced M1 inflammatory responses through JAK/STAT/SOCS signaling pathway. *TREM2* p.R47H mutation is also a risk factor for PD (Rayaprolu et al., 2013), and TREM2 level was upregulated in the midbrain of PD mice (Zhang Y. et al., 2018). TREM2 improves the phagocytosis ability of microglia, increases M2 marker Arg-1 and suppresses neuroinflammation in PD (Zhang Y. et al., 2018).

P2 × 7, a Purinergic P2 receptor for extracellular ATP (Volonté et al., 2011), deletion of which in SOD1^*G*93*A*^ mice shows a significantly faster and worse development of ALS, proving that P2 × 7 could reduce neuroinflammation (Apolloni et al., 2014). P2 × 7 antagonist Brilliant Blue G (BBG) significantly promotes motor neuron survival and reduces microgliosis in lumbar spinal cord at late pre-onset with reduced M1 markers and increased M2 markers (Apolloni et al., 2014).

## Cytockines Involved in Microglia Polarization in Neurodegenerative Diseases

Besides several cytokines mentioned above, milk fat globule epidermal growth factor 8 (MFG-E8) could reverse the increase of M1 markers and the decreased expression of M2 markers in Aβ_42_-treated microglia by down-regulating NF-κB and up-regulating PI3K-Akt pathways (Shi et al., 2017). IL-33, a newly described member of the IL-1 family, modulates the innate immune response by polarizing microglia toward M2 with enhanced Aβ phagocytic ability probably through down-regulating TLR signaling by MyD88 in APP/PS1 mice (Liu et al., 2010; Fu et al., 2016).

Wang et al. (2016) established scAAV9-VEGF-165 with cDNA of vascular endothelial growth factor (VEGF)-165 and delivered AAV9 virus by direct intrathecal injection. They found that scAAV9-VEGF-165 improved the motor function, prolonged the survival of SOD1^*G*93*A*^ mice, reduced M1 markers, and increased M2 markers with activation of the PI3K/AKT pathway and increased Bcl-2, which protects motor neurons (Wang et al., 2016).

## Ion Channel Involved in Microglia Polarization in Neurodegenerative Diseases

Microglia express high K_*v*_1.3 current densities when stimulated with LPS or a combination of LPS and IFN-γ (Nguyen et al., 2017), and K_*v*_1.3 is highly expressed in transgenic AD mouse models and AD patients’ brains (Maezawa et al., 2018). Maezawa et al. (2018) discovered that K_*v*_1.3 blocker PAP-1 could reduce neuroinflammation, decrease cerebral amyloid load, improve hippocampal neuronal plasticity, and improve behavioral deficits. Though they didn’t study the M1 and M2 phenotypes under the use of PAP-1, PAP-1 may probably shift microglia to M2.

Kir6.1-containing ATP-sensitive potassium (Kir6.1/K-ATP) channel skews microglia from M1 toward M2 phenotype by inhibiting p38 MAPK-NF-κB signaling pathway (Du et al., 2018). Knockout of Kir6.1 exaggerates inflammatory responses in the SNc and speeds up DA neuron degeneration in PD mice, while overexpression of Kir6.1 relieves toxic effects of M1 microglia (Du et al., 2018).

## Bioactive Compounds Inducing Microglia Polarization in Neurodegenerative Diseases

Low-density lipoprotein receptor related protein 1 (LRP-1) is the receptor of APOE and Aβ in the brain, contributing to Aβ clearance (Serrano-Pozo et al., 2021). Astaxanthin (ATX) could shift M1 microglia to M2 in a LRP-1-dependent manner by inhibiting NF-κB and JNK signaling, suggesting that ATX may be an effective therapeutic for AD by regulating microglia polarization (Wen et al., 2017). Dihydromyricetin (DHM) exerted neuroprotective effects in APP/PS1 mice, which significantly ameliorated memory and cognitive deficits, decreased activated microglia, reduced expression and activation of NLRP3 inflammasomes in APP/PS1 mice (Feng et al., 2018). DHM could promote clearance of Aβ by increasing levels of proteolytic enzyme neprilysin (NEP) and shifting microglia to M2 phenotype (Feng et al., 2018).

Ginsenoside Rg1 skews microglia from M1 toward M2 by inhibiting nuclear translocation of NF-κB (Liu et al., 2020), and exerts neuroprotective effect against inflammation-induced dopaminergic neuronal degeneration in SN in PD models (Sun et al., 2016). Capsaicin inhibits pro-inflammatory mediators, and shifts M1 microglia to M2, resulting in reduced oxidative damage and decreased degeneration of nigral dopamine neurons in the LPS-lesioned SN by transient receptor potential vanilloid 1 (TRPV1) (Bok et al., 2018), which is a capsaicin receptor and plays an important role in levodopa-induced dyskinesias (González-Aparicio and Moratalla, 2014). Ye et al. (2016) found that bee venom (BV)-derived phospholipase A2 (bvPLA2) significantly ameliorated motor dysfunction, down-regulated α-Syn, reduced the activation and numbers of microglia, and influenced microglia polarization to the M2 in human A53T α-Syn mutant transgenic (A53T Tg) mice. Camptothecin improves motor performance, reduces loss of neurons in the SN, and inhibits M1 polarization as well as promotes M2 polarization via the AKT/Nrf2/HO-1 and NF-κB signals in PD mice (He et al., 2021).

Protection of the BSCB integrity relieves clinical symptoms and demyelination in EAE (Zhang et al., 2020b). Tetramethylpyrazine (TMP) protects the BSCB integrity and alleviates EAE by decreasing microglia activation, and modulating microglia polarization from M1 to M2 through promoting STAT3/SOCS3 and inhibiting NF-κB signaling pathways (Zhang et al., 2020b).

## Drug Inducing Microglia Polarization in Neurodegenerative Diseases

Several studies found TLR4 increased in AD mice and in AD patients (Huang et al., 2017; Miron et al., 2018; Cui et al., 2020). TAK-242 (Ethyl (6R)-6-[N-(2-chloro-4-flfluorophenyl) sulfamoyl] cyclohex-1- ene-1-carboxylate), a specific small-molecule antagonist of TLR4, could polarize microglia from M1 to M2, and ameliorate learning and memory ability, lower Aβ deposition, and protect neuronal cells against cytotoxicity by down-regulating MyD88/NF-κB and NOD-like receptor family pyrin domain containing 3 (NLRP3, a key component in the inflammasome cascade) signaling in APP/PS1 mice (Cui et al., 2020). Candesartan could shift microglia toward a neuroprotective phenotype (Qie et al., 2020), and enhance phagocytosis of Aβ_1–42_ by microglia and significantly reduce amyloid burden and microglial activation in the hippocampus of AD mice (Torika et al., 2018). Guo et al. (2020) found that ROCK inhibitor Fasudi improved the cognitive deficits, inhibited microglial activation and promoted M2 phenotype in APP/PS1 mice by down-regulating TLR4/MyD88/NF-κB pathway.

Carta and Pisanu (2013) elaborated that PPAR-γ located in the SNc. PPAR-γ agonists exert a neuroprotective effect on PD by preventing the loss of DA neurons in the SNc (Carta, 2013). Pisanu et al. (2014) showed that PPAR-γ agonist rosiglitazone could switch microglia to M2 and had a helpful effect in 1-methyl-4-phenyl-1, 2, 3, 6-tetrahydropyridine (MPTP)-intoxicated mouse model of PD. Edaravone (3-methyl-1-phenyl-2-pyrazolin-5-one, EDA), an ROS scavenger, could mitigate motor dysfunction, inhibit LPS-induced microglial activation, remit declines of dopaminergic neurons in PD rats, possibly by inhibiting NLPR3 inflammasome activation and regulating microglia M1/M2 polarization (Li et al., 2021).

Microglia are sensitive to the histamine challenge mainly through histamine H1 receptors (Apolloni et al., 2016). H1 receptor antagonist Clemastine could reduce microgliosis, modulate microglia-related inflammatory genes, and improve motor neuron survival in SOD1^*G*93*A*^ mice (Apolloni et al., 2016). Clemastine induces increased P2Y12 and M2 marker Arg-1, and simultaneously inhibits M1 markers *in vitro* (Apolloni et al., 2016).

ROCK inhibitor Fasudil converts microglia from M1 to M2 by suppressing NF-κB, both *in vitro* and *in vivo*, thus attenuating demyelination and neuroinflammation, and promoting neuroprotection as well as reducing the severity of EAE (Zhang et al., 2013; Chen et al., 2014). Fingolimod (FTY720), a novel modulator of sphingosine 1-phosphate receptor (S1PR), has been approved by the US Food and Drug Administration as a first-line treatment for relapsing forms of multiple sclerosis (Brinkmann et al., 2010). Hu et al. (2021) found that FTY720 could significantly transform microglia from M1 to M2 through suppressing autophagy by STAT1 *in vitro*. FTY720 might also negatively modulate MAPK signaling pathway to inhibit neuroinflammation (Cipriani et al., 2015). Though no study *in vivo* has been performed, it’s likely that FTY720 could shift microglia phenotype from pro-inflammatory into anti-inflammatory in MS. Mitochondrial fission inhibitor 1 (Mdivi-1), an inhibitor of dynamin-related protein 1, decreased antigen presentation capacity of microglia and alleviated inflammation in EAE by polarizing microglia from M1 to M2 through inhibition of TLR2/4-activated GSK3β-NF-κB-p65 signaling pathway (Liu et al., 2021).

## Other Factors Inducing Microglia Polarization in Neurodegenerative Diseases

Mitochondrial oxidative stress is a key pathological factor in AD (Ren et al., 2020). Increased ROS levels switch microglia from M2 to M1 and induce AD, which is produced mainly in mitochondria by Nox, thus protecting mitochondria from oxidative stress would be useful to prevent and treat AD (Ren et al., 2020). Ren et al. (2020) synthesized TPP-MoS2 QDs, which mitigated Aβ aggregate-mediated neurotoxicity and removed Aβ aggregates in AD mice by regulating microglia polarization. AD patients have a high expression of NADPH oxidase in microglia (Choi et al., 2012), and inhibiting NADPH oxidase drives microglia phenotype from M1 to M2, which is a potential therapeutic approach in AD (Choi et al., 2012). Electroacupuncture, a widely used Chinese traditional therapy, improved learning, and memory of AD rats, inhibited the activation of glia and polarized microglia toward M2 phenotype in hippocampus of AD rats with decreased pro-inflammatory cytokines and increased anti-inflammatory cytokines by inhibiting NF-κB pathway but activating STAT-6 pathway (Xie et al., 2021). Over-expression of thymosin β4 (Tβ4), a constituent part of cytoplasm and cytoskeleton, improved neuronal function and cognitive ability, reduced brain Aβ accumulation, upregulated insulin-degrading enzyme, and alleviated neuroinflammatory response by converting microglia and astrocyte to neuroprotective phenotype in APP/PS1 mice via negative regulation of TLR4/MyD88/NF-κB p65 and p52 pathways (Wang et al., 2021).

Epigenetic modification can regulate gene expression by histone post-translational modification or changing DNA methylation (Berger, 2007). Jumonji domain containing 3 (Jmjd3), a histone H3K27me3 demethylase, is essential for the epigenetic modulation of M2 polarization depending on its demethylase activity to modify histone H3K27me3 (Tang et al., 2014). Suppression of Jmjd3 inhibits M2 polarization and simultaneously exaggerates inflammatory responses, which lead to extensive neuron death (Tang et al., 2014). M2 marker Arg1, a direct downstream target of Jmjd3, could partly reduce the harmful effects and exert neuroprotection effects as Jmjd3 is suppressed, acting as a critical neuron-survival molecule (Tang et al., 2014). Long non-coding RNAs (lncRNAs) can regulate the function of target genes through epigenetic mechanisms as well, and are involved in the progression of neurodegenerative diseases (Yang et al., 2021). HOXA cluster antisense RNA 2 (HOXA-AS2), one of the most abundantly expressed lncRNAs, was found upregulated in PD patients (Yang et al., 2021). Upregulated HOXA-AS2 promotes neuroinflammation by regulating microglia polarization through interacting with polycomb repressive complex 2 (PRC2) and epigenetically silencing PGC-1α in PD. HOXA-AS2 knockdown could significantly repress microglia M1 polarization and promote M2 polarization (Yang et al., 2021).

TLR2 signaling takes part in the inflammatory response and inhibits remyelination in MS (Esser et al., 2018). Wasko et al. (2019) found that TLR2 tolerance during remyelination significantly improved myelin recovery, associated with a shift in corpus callosum microglia from M1 to M2 phenotype. IRAK-M increases significantly in EAE, and transforms M1 microglia into M2 phenotype by inhibiting NF-κB and STAT1 (Liu et al., 2019a). IRAK-M also reduces the incidence of EAE and relieves clinical symptoms (Liu et al., 2019a). 2-arachidonoylglycerol (2AG) could significantly delay the onset of EAE, and reduce relapse severity, chronic disability and mortality in EAE, which may result from its function to balance the inflammatory environment in the CNS, to increase microglial activation, and to shift microglia to M2 phenotype (Lourbopoulos et al., 2011). The homeobox gene msh-like homeobox-3 (Msx3), the third member of the murine Msx homeobox gene family, affects the development of the dorsal neural tube (Shimeld et al., 1996). Abnormal MSX3 expression in microglia contributes to demyelination and neurodegeneration in EAE (Yu et al., 2015). While overexpression of MSX3 in microglia promotes the survival, differentiation and neurite growth of oligodendrocyte progenitor cells, which produce myelin, through polarizing M1 microglia toward M2 by PPAR-γ and JAK/STAT6 pathways (Yu et al., 2015). Aryanpour et al. (2017) found that PRO decreased neurobehavioral defects, improved remyelination, reduced neuroinflammation, and shifted microglia from M1 to M2 in a cuprizone (CPZ)-induced demyelination mouse model. Exogenous 17β-estradiol therapy leads to the reduction of M1 phenotype, stimulation of polarized M2 microglia, and repression of NLRP3 inflammasome in the corpus callosum of CPZ demyelination model of MS (Aryanpour et al., 2021). It also improves neurological behavioral deficits, and causes a decrease in demyelination levels and axonal injury, seeming to facilitate and accelerate the remyelination process (Aryanpour et al., 2021). Exosomes are a type of extracellular vesicle that contain constituents (proteins, metabolites, and nucleic acids) of the cells secreting them. They are taken up by distant cells, and constituents of exosomes could effectively affect cell function and behavior, which can be disease promoting or restraining (Kalluri and LeBleu, 2020). Intercellular communication through exosomes seems to be involved in immune responses, viral pathogenicity, pregnancy, cardiovascular diseases, central nervous system–related diseases, and cancer progression (Kalluri and LeBleu, 2020). Bone marrow mesenchymal stem cell (BMSC)-derived exosome significantly reduces inflammation and demyelination in the CNS, attenuates clinical manifestation of EAE, and significantly increases M2 markers and decreases M1 markers (Li et al., 2019). Zhang et al. (2021) also found that exosomes released from mesenchymal stromal cells (MSC-Exo) significantly improved neurological outcome, increased the numbers oligodendrocytes and the level of myelin basic protein, and decreased amyloid-β precursor protein density in EAE and CPZ models of demyelination. MSC-Exo also decreased neuroinflammation by increasing M2 phenotype and decreasing M1 phenotype of microglia via inhibition of the TLR2/IRAK1/NF-κB signaling pathway (Zhang et al., 2021).

## Conclusion

**FIGURE 2:**
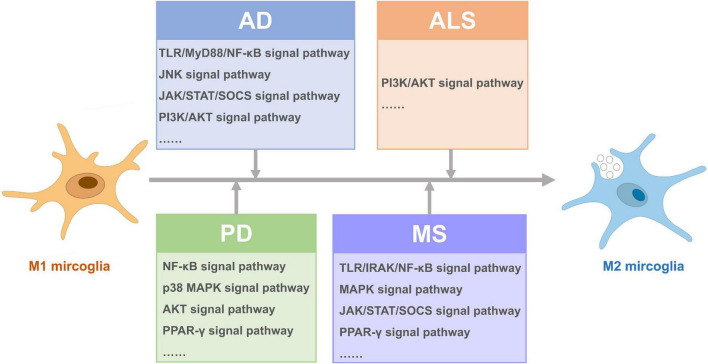
Signal pathways activated for microglia polarization from M1 to M2 in different neurodegenerative diseases.

In this review, we briefly introduced the function and phenotype of microglia and focused on the role and polarization regulation of microglia in the pathogenesis of neurodegenerative diseases, including AD, PD, ALS, and MS, to probe into potential therapeutic strategies for neurodegenerative diseases. The modulators in microglia polarization from M1 to M2 can be divided into several categories, including transcription factors, receptors, cytokines, ion channels, bioactive compounds, and drugs (Supplementary Table 1), most of which are related to several important signal pathways (Figure 2). It seems transcription factors, such as NF-κB, may be the key factors in these important signal pathways to shift microglia from M1 to M2. Nowadays, most of the compounds applied in neurodegenerative diseases are designed to suppress neuroinflammation through simply inhibiting M1 phenotype, and a few compounds have been verified to shift microglia polarization to M2 phenotype (Zhang B. et al., 2018). However, recent studies have shown that inhibiting M1 microglia alone is not enough to achieve therapeutic significance in neurodegenerative diseases (Becker et al., 2011; Miguel-Álvarez et al., 2015; Du et al., 2018). It is worth noting that specific neurodegenerative genotypes may affect the effects of anti-inflammatory drugs in neurodegenerative diseases, for example, regular use of non-steroidal anti-inflammatory drugs can reduce the risk of PD among leucine rich repeat kinase 2 (LRRK2) variant carriers, which causes the most common Mendelian genetic form of PD (San Luciano et al., 2020). Nevertheless, inhibiting M1 microglia and promoting M2 microglial activation at the same time is required for therapy (Yang et al., 2019). Thus, further researches are needed to reveal the marked mystery in microglia polarization. It is worth noting that these studies in the specific disease environment of neurodegenerative diseases are obviously less than those *in vitro* or in other diseases like ischemic stroke, spinal cord injury, and traumatic brain injury, which may have implications for further researches. Nevertheless, whether these mechanisms in these diseases and wild type mice or *in vivo* could be analogy to neurodegenerative diseases is unknown. What’s more, some mechanisms of microglia polarization modulation are the same *in vitro* and *in vivo* of individuals with neurodegenerative diseases, such as PPAR-γ agonist (Pisanu et al., 2014; Yang et al., 2019), IL-4 and so on, while other mechanisms verified *in vitro* are not applicable in neurodegenerative diseases, which needs further research. In addition, different regulators in microglia phenotype may be related to one another. For example, IL-33 skews microglia phenotype from M2 to M1 by down-regulating TLR signaling by MyD88 pathways (Fu et al., 2016), which links cytokine and signal pathways. And many bioactive compounds and drugs change the phenotype of microglia by inhibiting or promoting important signal pathways as well, suggesting the important role of signal pathways in microglia polarization. It seems the mechanisms of microglia polarization are complex, and combining different mechanisms together may have a helpful effect in the therapy of neurodegenerative diseases. As specific neurodegenerative genotypes affect the effects of anti-inflammatory drugs in neurodegenerative diseases, whether neurodegenerative disease-related risk genes may affect microglia polarization deserves to be discussed. For example, *TREM2* (rs75932628, p.R47H) mutation is a risk factor of AD and PD, and TREM2 promotes M2 microglia polarization (Song and Suk, 2017; Zhang B. et al., 2018). The relationship between other risk genes and microglia polarization in neurodegenerative diseases remains to be studied.

## Author Contributions

SG wrote the manuscript. YY and HW reviewed and revised the manuscript. All authors contributed to the article and approved the submitted version.

## Conflict of Interest

The authors declare that the research was conducted in the absence of any commercial or financial relationships that could be construed as a potential conflict of interest.

## Publisher’s Note

All claims expressed in this article are solely those of the authors and do not necessarily represent those of their affiliated organizations, or those of the publisher, the editors and the reviewers. Any product that may be evaluated in this article, or claim that may be made by its manufacturer, is not guaranteed or endorsed by the publisher.
