# Supplementary material for: Microglia Polarization From M1 to M2 in Neurodegenerative Diseases
Source: Front Aging Neurosci. 2022 Feb 16;14:815347. doi: 10.3389/fnagi.2022.815347 (PMC8888930; doi:10.3389/fnagi.2022.815347)
Supplement: Supplementary file 1 [file Table_1.docx]

**Supplementary Table 1. Modulators in microglia polarization from M1 to M2**

| **Classification** | **Modulators** | **Related signaling pathways** | **Disease** | **Effects** | **Reference** |
| --- | --- | --- | --- | --- | --- |
| Transcription factor | **NF-κB** | - | - | Suppressed the inflammatory gene and drove microglia to M2 | (1) |
|  | **PPAR-γ** | LKB1/AMPK pathway | - | Promoted M1 to M2 through improving autophagy | (2) |
| Receptors | **TREM2** | - | - | Promoted M2 polarization and reduced microglia-mediated inflammation | (3) |
|  |  | JAK/STAT/SOCS pathway | AD | Rescued cognitive deficits, decreased Aβ plaques deposition, reduced synaptic and neuronal loss, ameliorated neuroinflammation, promoted M2 polarization, and reduced M1 inflammatory responses | (4) |
|  |  | - | PD | Improved the phagocytosis ability of microglia, increased M2 marker Arg-1 and suppressed neuroinflammation | (5) |
|  | **Acetylcholine** | JAK2/STAT3 pathway | - | Transformed M1 microglia to M2 through promoting α7 subtype of the nicotinic acetylcholine receptor (α7 nAChR) | (6) |
|  | **P2X7 (Purinergic P2 receptor for extracellular ATP)** | - | ALS | Reduced neuroinflammation, promoted motor neuron to survive and reduced microgliosis in lumbar spinal cord at late pre-onset with reduced M1 markers and increased M2 markers | (7) |
| Cytokines | **IL-4** | - | - | Shifted microglia from M1 towards M2 | (8) |
|  | **Insulin-like growth factor-1** | - | - | Shifted microglia from M1 towards M2 | (9) |
|  | **Glial cell-derived neurotrophic factor** | PI3K/AKT pathway | - | Exerted neuroprotective effects, and modulated microglial polarization to M2 phenotype | (10) |
|  | **Milk fat globule epidermal growth factor 8** | NF-κB and  PI3K/Akt pathways | AD | Reversed the increased M1 markers and the  decreased M2 markers | (11) |
|  | **IL-33** | TLR/MyD88 pathways | AD | Modulated the innate immune response by polarizing microglia toward M2 with enhanced Aβ phagocytic ability | (12) |
|  | **scAAV9-VEGF-165** | PI3K/Akt pathway | ALS | Improved the motor function, prolonged the survival of SOD1G93A mice, reduced M1 markers, and increased M2 markers | (13) |
| Ion channels | **Kv 1.3 blocker PAP-1** | - | AD | Reduced neuroinflammation, decreased cerebral amyloid load, improved hippocampal neuronal plasticity, and improved behavioral deficits | (14) |
|  | **Kir6.1/K-ATP channel** | p38 MAPK-NF-κB pathway | PD | Skewed microglia from M1 toward M2, and relieved toxic effects of M1 microglia | (15) |
| Bioactive compounds | **Astaxanthin** | NF-κB and JNK pathways | - | Shifted microglia from M1 to M2 | (16) |
|  | **Anisalcohol** | NF-κB and JNK pathways | - | Shifted microglia from M1 to M2 | (17) |
|  | **Naringenin** | MAPK, especially JNK pathway | - | Shifted microglia from M1 to M2 | (18) |
|  | **Resveratrol** | PGC-1α pathway  NF-κB pathway STAT6 and STAT3 pathways | - | Shifted microglia from M1 to M2 | (19) |
|  | **Platycodigenin** | NF-κB pathway | - | Shifted microglia from M1 to M2 | (20) |
|  | **Hydroxytyrosol** | NF-κB and ERK pathway | - | Shifted microglia from M1 to M2 | (21) |
|  | **Curcumin** | TLR4/NF-κB pathway | - | Shifted microglia from M1 to M2 | (22) |
|  |  | CaMKKβ-dependent AMPK pathway | - | Shifted microglia from M1 to M2 | (23) |
|  |  | JAK/STAT/SOCS pathway | - | Elicited anti-inflammatory responses | (24) |
|  | **Isovitexin** | CaMKKβ/AMPK pathway | - | Shifted microglia from M1 to M2 | (25) |
|  | **Betulinic acid** | CaMKKβ/AMPK pathway | - | Shifted microglia from M1 to M2 | (26) |
|  | **Rosmarinic acid** | PDPK1/Akt/HIF pathway | - | Shifted microglia from M1 to M2 | (27) |
|  | **Dihydromyricetin** | - | AD | Ameliorated memory and cognitive deficits, decreased activated microglia, reduced expression and activation of NLRP3 inflammasomes, and promoted clearance of Aβ by increasing levels of  proteolytic enzyme neprilysin and shifting microglia to M2 phenotype | (28) |
|  | **Ginsenoside Rg1** | NF-κB pathway | PD | Skewed microglia from M1 toward M2, and exerted neuroprotective effect against inflammation-induced dopaminergic neuronal degeneration in SN | (29) |
|  | **Capsaicin** | - | PD | Inhibited pro-inflammatory mediators, shifted the M1 microglia to M2, and reduced oxidative damage and degeneration of nigral dopamine neurons in the LPS-lesioned SN | (30) |
|  | **Bee venom -derived phospholipase A2** | - | PD | Ameliorated motor dysfunction, down-regulated α-Syn, reduced the activation and numbers of microglia, and influenced microglia polarization to the M2 phenotype | (31) |
|  | **Camptothecin** | AKT/Nrf2/HO-1 and NF-κB pathways | PD | Improved motor performance, reduced the loss of neurons in the SN, and inhibited M1 polarization and promoted M2 polarization | (32) |
|  | **Tetramethylpyrazine** | STAT3/SOCS3 and NF-кB pathways | EAE/MS | Protected the BSCB integrity, and alleviates EAE by decreasing microglia activation and modulating microglia polarization from M1 to M2 | (33) |
| Drugs | **Fasudil** | Rho/ROCK pathway | - | Reduced pro-inflammatory factors NO, IL-1β, IL-6 and TNFα and increased anti-inflammatory factor IL-10 | (34) |
|  |  | NF-κB pathway | - | Led to a M2 phenotype | (35) |
|  |  | TLR4/Myd88/NF-κB pathway | AD | Improved the cognitive deficits, inhibited microglial activation and promoted M2 phenotype | (36) |
|  |  | NF-κB pathway | EAE/MS | Converted microglia from M1 to M2, attenuated demyelination and neuroinflammation, promoted neuroprotection, and reduced the severity of EAE | (37) |
|  | **Candesartan(angiotensin II type I receptor blocker)** | TLR4/NF-κB  pathway | - | Modulated the neuroinflammatory response, reversed neurotoxic effect, and shifted microglia from M1 to M2 | (38) |
|  |  |  | AD | Shift microglia toward M2 phenotype, enhanced phagocytosis of Aβ1-42 by microglia, and significantly reduced amyloid burden and microglial activation | (39) |
|  | **Telmisartan(angiotensin II type I receptor blocker)** | PPAR-γ and CaMKKβ/AMPK pathway | - | Promoted M2 polarization and reduced M1 polarization | (40) |
|  | **Dexmedetomidine(alpha2 adrenoceptor agonist)** | ERK1/2 pathway | - | Promoted microglial M2 polarization | (41) |
|  | **Simvastatin(hydroxymethylglutaryl–coenzyme A reductase inhibitor)** | Notch pathway | - | Regulated microglia polarization from M1 to M2 | (42) |
|  | **Nicotine** | STAT3 pathway | - | Promoted M2 microglial polarization | (43) |
|  | **TAK-242(TLR4 antagonist)** | MyD88/NF-κB pathway | AD | Induced microglia switch from M1 to M2, ameliorated learning and memory ability, lowered Aβ deposition, and protected neuronal cells against cytotoxicity | (44) |
|  | **Rosiglitazone(PPAR-γ agonist)** | - | PD | Switched microglia to M2 | (45) |
|  | **Edaravone(ROS scavenger)** | - | PD | Mitigated motor dysfunction, inhibited LPS-induced microglial activation, remitted declines of dopaminergic neurons, possibly by inhibiting NLPR3 inflammasome activation and regulating microglia M1/M2 polarization | (46) |
|  | **Clemastine (histamine H1 receptor antagonist)** |  | ALS | Reduced microgliosis, modulated microglia-related inflammatory genes, improved motor neuron survival, and induced increased P2Y12 and M2 marker Arg-1, and simultaneous inhibited M1 markers | (47) |
|  | **Fingolimod (modulator of sphingosine 1-phosphate receptor)** | STAT1 pathway | - | Transformed microglia from M1 to M2 through suppressing autophagy | (48) |
| Others | **NADPH oxidase/ROS** | - | - | Inhibiting NADPH oxidase drives microglia phenotype from M1 to M2 | (49) |
|  | **Mitochondrial fission inhibitor 1 (Mdivi-1)** | TLR2/4-activated GSK3β-NF-κB-p65 pathway | EAE | Decreased antigen presentation capacity of microglia and alleviated inflammation by polarization of microglia from M1 to M2 phenotype | (50) |
|  | **CDGSH iron-sulfur domain 2** | NF-κB pathway | - | Exerted anti-inflammatory effects and preserved M2 microglia | (51) |
|  | **Triglyceride-rich lipoprotein (TRL)** | - | - | Postprandial TRL-monounsaturated fatty acids promote M2 microglia polarization, whereas postprandial TRL-saturated fatty acids improve M1 microglia polarization | (52) |
|  | **Melatonin** | melatonin receptor 1/JAK2/STAT3/telomerase pathway | - | Ameliorated neurobehavioral disturbances, alleviated axonal hypomyelination in the periventricular white matter of LPS-injected postnatal rats, and reduces neuroinflammation through shifting M1 microglia towards M2 | (53) |
|  | **TPP-MoS2 QDs** | - | AD | Mitigated Aβ aggregate-mediated neurotoxicity and removed Aβ aggregates by switching microglia phenotype | (54) |
|  | **Electroacupuncture** | NF-κB & STAT-6 pathway. | AD | Improved learning and memory inhibited the activation of glia and polarized microglia toward the M2 phenotype in hippocampus with decreased pro-inflammatory cytokines and increased anti-inflammatory cytokines | (55) |
|  | **Thymosin β4 (Tβ4)** | TLR4/MyD88/NF-κB p65 and p52 pathways | AD | Improved neuronal function and cognitive ability, reduced brain Aβ accumulation, upregulated insulin-degrading enzyme, and alleviated neuroinflammatory response by converting microglia and astrocyte to neuroprotective phenotype | (56) |
|  | **Jumonji domain containing 3 (Jmjd3)** | - | PD | Suppression of Jmjd3 inhibited M2 polarization and simultaneously exaggerated inflammatory responses. | (57) |
|  | **HOXA cluster antisense RNA 2 (HOXA-AS2)** | - | PD | HOXA-AS2 knockdown could significantly repress microglial M1 polarization and promote M2 polarization | (58) |
|  | **TLR2 tolerance** | TLR2 pathway | - | Improved myelin recovery, and shifted microglia from M1 to M2 phenotype | (59) |
|  | **2-arachidonoylglycerol** | - | EAE | Delayed the onset of EAE, reduced relapse severity, chronic disability and mortality in EAE, increased microglial activation, and shifted microglia to M2 phenotype | (60) |
|  | **The homeobox gene msh-like homeobox-3** | PPAR-γ & JAK/STAT6 pathways | EAE | Contributed to demyelination and neurodegeneration in EAE, promoted the survival, differentiation and neurite growth of oligodendrocyte progenitor cells through promoting M1 microglia to polarize towards M2 | (61) |
|  | **17β-estradiol** | - | CPZ | Led to the reduction of M1 phenotype, stimulation of polarized M2 microglia, and repression of NLRP3 inflammasome in the corpus callosum of CPZ demyelination model of MS, improved neurological behavioral deficits, and caused a decrease in demyelination levels and axonal injury | (62) |
|  | **Bone marrow mesenchymal stem cell-derived exosome** | - | EAE | Reduced inflammation and demyelination in the CNS, attenuated clinical manifestation of EAE, increased M2 markers, and decreased M1 markers | (63) |
|  | **Exosomes released from mesenchymal stromal cells (MSC-Exo)** | TLR2/IRAK1/NF-κB pathway | EAE&CPZ | Improved neurological outcome, increased the numbers oligodendrocytes and the level of myelin basic protein, decreased amyloid-β precursor protein density, and decreased neuroinflammation by increasing the M2 phenotype and decreasing the M1 phenotype of microglia, as well as their related cytokines and special markers | (64) |

1. Zhang H, Li Y, Yu J, Guo M, Meng J, Liu C, et al. Rho kinase inhibitor fasudil regulates microglia polarization and function. *Neuroimmunomodulation* (2013) 20(6):313-22. Epub 2013/08/15. doi: 10.1159/000351221. PubMed PMID: 23941863.

2. Ji J, Xue TF, Guo XD, Yang J, Guo RB, Wang J, et al. Antagonizing peroxisome proliferator-activated receptor gamma facilitates M1-to-M2 shift of microglia by enhancing autophagy via the LKB1-AMPK signaling pathway. *Aging Cell* (2018) 17(4):e12774. Epub 2018/05/10. doi: 10.1111/acel.12774. PubMed PMID: 29740932; PubMed Central PMCID: PMCPMC6052482.

3. Sekiyama K, Sugama S, Fujita M, Sekigawa A, Takamatsu Y, Waragai M, et al. Neuroinflammation in Parkinson's Disease and Related Disorders: A Lesson from Genetically Manipulated Mouse Models of α-Synucleinopathies. *Parkinsons Dis* (2012) 2012:271732. Epub 2012/05/03. doi: 10.1155/2012/271732. PubMed PMID: 22550610; PubMed Central PMCID: PMCPMC3324936.

4. Ruganzu JB, Zheng Q, Wu X, He Y, Peng X, Jin H, et al. TREM2 overexpression rescues cognitive deficits in APP/PS1 transgenic mice by reducing neuroinflammation via the JAK/STAT/SOCS signaling pathway. *Exp Neurol* (2021) 336:113506. Epub 2020/10/17. doi: 10.1016/j.expneurol.2020.113506. PubMed PMID: 33065077.

5. Zhang Y, Feng S, Nie K, Li Y, Gao Y, Gan R, et al. TREM2 modulates microglia phenotypes in the neuroinflammation of Parkinson's disease. *Biochem Biophys Res Commun* (2018) 499(4):797-802. Epub 2018/04/06. doi: 10.1016/j.bbrc.2018.03.226. PubMed PMID: 29621548.

6. Zhang Q, Lu Y, Bian H, Guo L, Zhu H. Activation of the α7 nicotinic receptor promotes lipopolysaccharide-induced conversion of M1 microglia to M2. *Am J Transl Res* (2017) 9(3):971-85. Epub 2017/04/08. PubMed PMID: 28386326; PubMed Central PMCID: PMCPMC5375991.

7. Apolloni S, Amadio S, Parisi C, Matteucci A, Potenza RL, Armida M, et al. Spinal cord pathology is ameliorated by P2X7 antagonism in a SOD1-mutant mouse model of amyotrophic lateral sclerosis. *Dis Model Mech* (2014) 7(9):1101-9. Epub 2014/07/20. doi: 10.1242/dmm.017038. PubMed PMID: 25038061; PubMed Central PMCID: PMCPMC4142730.

8. Tang RH, Qi RQ, Liu HY. Interleukin-4 affects microglial autophagic flux. *Neural Regen Res* (2019) 14(9):1594-602. Epub 2019/05/16. doi: 10.4103/1673-5374.255975. PubMed PMID: 31089059; PubMed Central PMCID: PMCPMC6557092.

9. Labandeira-Garcia JL, Costa-Besada MA, Labandeira CM, Villar-Cheda B, Rodriguez-Perez AI. Insulin-Like Growth Factor-1 and Neuroinflammation. *Front Aging Neurosci* (2017) 9:365. Epub 2017/11/23. doi: 10.3389/fnagi.2017.00365. PubMed PMID: 29163145; PubMed Central PMCID: PMCPMC5675852.

10. Zhong Z, Chen A, Fa Z, Ding Z, Xie J, Sun Y, et al. Adipose-Derived Stem Cells Modulate BV2 Microglial M1/M2 Polarization by Producing GDNF. *Stem Cells Dev* (2020) 29(11):714-27. Epub 2020/03/01. doi: 10.1089/scd.2019.0235. PubMed PMID: 32111146.

11. Shi X, Cai X, Di W, Li J, Xu X, Zhang A, et al. MFG-E8 Selectively Inhibited Abeta-Induced Microglial M1 Polarization via NF-kappaB and PI3K-Akt Pathways. *Mol Neurobiol* (2017) 54(10):7777-88. Epub 2016/11/16. doi: 10.1007/s12035-016-0255-y. PubMed PMID: 27844286.

12. Fu AK, Hung KW, Yuen MY, Zhou X, Mak DS, Chan IC, et al. IL-33 ameliorates Alzheimer's disease-like pathology and cognitive decline. *Proc Natl Acad Sci U S A* (2016) 113(19):E2705-13. Epub 2016/04/20. doi: 10.1073/pnas.1604032113. PubMed PMID: 27091974; PubMed Central PMCID: PMCPMC4868478.

13. Wang Y, Duan W, Wang W, Di W, Liu Y, Liu Y, et al. scAAV9-VEGF prolongs the survival of transgenic ALS mice by promoting activation of M2 microglia and the PI3K/Akt pathway. *Brain Res* (2016) 1648(Pt A):1-10. Epub 2016/07/10. doi: 10.1016/j.brainres.2016.06.043. PubMed PMID: 27392886.

14. Maezawa I, Nguyen HM, Di Lucente J, Jenkins DP, Singh V, Hilt S, et al. Kv1.3 inhibition as a potential microglia-targeted therapy for Alzheimer's disease: preclinical proof of concept. *Brain* (2018) 141(2):596-612. Epub 2017/12/23. doi: 10.1093/brain/awx346. PubMed PMID: 29272333; PubMed Central PMCID: PMCPMC5837198.

15. Du RH, Sun HB, Hu ZL, Lu M, Ding JH, Hu G. Kir6.1/K-ATP channel modulates microglia phenotypes: implication in Parkinson's disease. *Cell Death Dis* (2018) 9(3):404. Epub 2018/03/16. doi: 10.1038/s41419-018-0437-9. PubMed PMID: 29540778; PubMed Central PMCID: PMCPMC5852118.

16. Wen X, Xiao L, Zhong Z, Wang L, Li Z, Pan X, et al. Astaxanthin acts via LRP-1 to inhibit inflammation and reverse lipopolysaccharide-induced M1/M2 polarization of microglial cells. *Oncotarget* (2017) 8(41):69370-85. Epub 2017/10/21. doi: 10.18632/oncotarget.20628. PubMed PMID: 29050210; PubMed Central PMCID: PMCPMC5642485.

17. Xiang B, Xiao C, Shen T, Li X. Anti-inflammatory effects of anisalcohol on lipopolysaccharide-stimulated BV2 microglia via selective modulation of microglia polarization and down-regulation of NF-kappaB p65 and JNK activation. *Mol Immunol* (2018) 95:39-46. Epub 2018/02/07. doi: 10.1016/j.molimm.2018.01.011. PubMed PMID: 29407575.

18. Zhang B, Wei YZ, Wang GQ, Li DD, Shi JS, Zhang F. Targeting MAPK Pathways by Naringenin Modulates Microglia M1/M2 Polarization in Lipopolysaccharide-Stimulated Cultures. *Front Cell Neurosci* (2018) 12:531. Epub 2019/01/29. doi: 10.3389/fncel.2018.00531. PubMed PMID: 30687017; PubMed Central PMCID: PMCPMC6336899.

19. Yang X, Xu S, Qian Y, Xiao Q. Resveratrol regulates microglia M1/M2 polarization via PGC-1alpha in conditions of neuroinflammatory injury. *Brain Behav Immun* (2017) 64:162-72. Epub 2017/03/08. doi: 10.1016/j.bbi.2017.03.003. PubMed PMID: 28268115.

20. Yang Z, Liu B, Yang LE, Zhang C. Platycodigenin as Potential Drug Candidate for Alzheimer's Disease via Modulating Microglial Polarization and Neurite Regeneration. *Molecules* (2019) 24(18). Epub 2019/09/07. doi: 10.3390/molecules24183207. PubMed PMID: 31487775; PubMed Central PMCID: PMCPMC6767002.

21. Zhang L, Zhang J, Jiang X, Yang L, Zhang Q, Wang B, et al. Hydroxytyrosol Inhibits LPS-Induced Neuroinflammatory Responses via Suppression of TLR-4-Mediated NF-kappaB P65 Activation and ERK Signaling Pathway. *Neuroscience* (2020) 426:189-200. Epub 2019/12/24. doi: 10.1016/j.neuroscience.2019.12.005. PubMed PMID: 31866556.

22. Zhang J, Zheng Y, Luo Y, Du Y, Zhang X, Fu J. Curcumin inhibits LPS-induced neuroinflammation by promoting microglial M2 polarization via TREM2/ TLR4/ NF-kappaB pathways in BV2 cells. *Mol Immunol* (2019) 116:29-37. Epub 2019/10/08. doi: 10.1016/j.molimm.2019.09.020. PubMed PMID: 31590042.

23. Qiao P, Ma J, Wang Y, Huang Z, Zou Q, Cai Z, et al. Curcumin Prevents Neuroinflammation by Inducing Microglia to Transform into the M2-phenotype via CaMKKbeta-dependent Activation of the AMP-Activated Protein Kinase Signal Pathway. *Curr Alzheimer Res* (2020) 17(8):735-52. Epub 2020/11/13. doi: 10.2174/1567205017666201111120919. PubMed PMID: 33176649.

24. Porro C, Cianciulli A, Trotta T, Lofrumento DD, Panaro MA. Curcumin Regulates Anti-Inflammatory Responses by JAK/STAT/SOCS Signaling Pathway in BV-2 Microglial Cells. *Biology (Basel)* (2019) 8(3). Epub 2019/06/30. doi: 10.3390/biology8030051. PubMed PMID: 31252572; PubMed Central PMCID: PMCPMC6784227.

25. Liu B, Huang B, Hu G, He D, Li Y, Ran X, et al. Isovitexin-Mediated Regulation of Microglial Polarization in Lipopolysaccharide-Induced Neuroinflammation via Activation of the CaMKKbeta/AMPK-PGC-1alpha Signaling Axis. *Front Immunol* (2019) 10:2650. Epub 2019/12/05. doi: 10.3389/fimmu.2019.02650. PubMed PMID: 31798583; PubMed Central PMCID: PMCPMC6868066.

26. Li C, Zhang C, Zhou H, Feng Y, Tang F, Hoi MPM, et al. Inhibitory Effects of Betulinic Acid on LPS-Induced Neuroinflammation Involve M2 Microglial Polarization via CaMKKbeta-Dependent AMPK Activation. *Front Mol Neurosci* (2018) 11:98. Epub 2018/04/19. doi: 10.3389/fnmol.2018.00098. PubMed PMID: 29666569; PubMed Central PMCID: PMCPMC5891622.

27. Wei Y, Chen J, Cai GE, Lu W, Xu W, Wang R, et al. Rosmarinic Acid Regulates Microglial M1/M2 Polarization via the PDPK1/Akt/HIF Pathway Under Conditions of Neuroinflammation. *Inflammation* (2021) 44(1):129-47. Epub 2020/09/18. doi: 10.1007/s10753-020-01314-w. PubMed PMID: 32940818.

28. Feng J, Wang JX, Du YH, Liu Y, Zhang W, Chen JF, et al. Dihydromyricetin inhibits microglial activation and neuroinflammation by suppressing NLRP3 inflammasome activation in APP/PS1 transgenic mice. *CNS Neurosci Ther* (2018) 24(12):1207-18. Epub 2018/06/06. doi: 10.1111/cns.12983. PubMed PMID: 29869390; PubMed Central PMCID: PMCPMC6282966.

29. Liu JQ, Zhao M, Zhang Z, Cui LY, Zhou X, Zhang W, et al. Rg1 improves LPS-induced Parkinsonian symptoms in mice via inhibition of NF-kappaB signaling and modulation of M1/M2 polarization. *Acta Pharmacol Sin* (2020) 41(4):523-34. Epub 2020/03/24. doi: 10.1038/s41401-020-0358-x. PubMed PMID: 32203085; PubMed Central PMCID: PMCPMC7468333.

30. Bok E, Chung YC, Kim KS, Baik HH, Shin WH, Jin BK. Modulation of M1/M2 polarization by capsaicin contributes to the survival of dopaminergic neurons in the lipopolysaccharide-lesioned substantia nigra in vivo. *Exp Mol Med* (2018) 50(7):1-14. Epub 2018/07/04. doi: 10.1038/s12276-018-0111-4. PubMed PMID: 29968707; PubMed Central PMCID: PMCPMC6030094.

31. Ye M, Chung H-S, Lee C, Hyun Song J, Shim I, Kim Y-S, et al. Bee venom phospholipase A2 ameliorates motor dysfunction and modulates microglia activation in Parkinson’s disease alpha-synuclein transgenic mice. *Exp Mol Med* (2016) 48(7):e244-e. doi: 10.1038/emm.2016.49.

32. He D, Fu S, Zhou A, Su Y, Gao X, Zhang Y, et al. Camptothecin Regulates Microglia Polarization and Exerts Neuroprotective Effects via Activating AKT/Nrf2/HO-1 and Inhibiting NF-kappaB Pathways In Vivo and In Vitro. *Front Immunol* (2021) 12:619761. Epub 2021/04/20. doi: 10.3389/fimmu.2021.619761. PubMed PMID: 33868235; PubMed Central PMCID: PMCPMC8047064.

33. Zhang L, Lu X, Gong L, Cui L, Zhang H, Zhao W, et al. Tetramethylpyrazine Protects Blood-Spinal Cord Barrier Integrity by Modulating Microglia Polarization Through Activation of STAT3/SOCS3 and Inhibition of NF-small ka, CyrillicB Signaling Pathways in Experimental Autoimmune Encephalomyelitis Mice. *Cell Mol Neurobiol* (2020). Epub 2020/05/20. doi: 10.1007/s10571-020-00878-3. PubMed PMID: 32424774.

34. Koch JC, Tatenhorst L, Roser AE, Saal KA, Tonges L, Lingor P. ROCK inhibition in models of neurodegeneration and its potential for clinical translation. *Pharmacol Ther* (2018) 189:1-21. Epub 2018/04/06. doi: 10.1016/j.pharmthera.2018.03.008. PubMed PMID: 29621594.

35. Chen J, Sun Z, Jin M, Tu Y, Wang S, Yang X, et al. Inhibition of AGEs/RAGE/Rho/ROCK pathway suppresses non-specific neuroinflammation by regulating BV2 microglial M1/M2 polarization through the NF-κB pathway. *J Neuroimmunol* (2017) 305:108-14. Epub 2017/03/13. doi: 10.1016/j.jneuroim.2017.02.010. PubMed PMID: 28284330.

36. Guo MF, Zhang HY, Li YH, Gu QF, Wei WY, Wang YY, et al. Fasudil inhibits the activation of microglia and astrocytes of transgenic Alzheimer's disease mice via the downregulation of TLR4/Myd88/NF-kappaB pathway. *J Neuroimmunol* (2020) 346:577284. Epub 2020/07/12. doi: 10.1016/j.jneuroim.2020.577284. PubMed PMID: 32652366.

37. Chen C, Li YH, Zhang Q, Yu JZ, Zhao YF, Ma CG, et al. Fasudil regulates T cell responses through polarization of BV-2 cells in mice experimental autoimmune encephalomyelitis. *Acta Pharmacol Sin* (2014) 35(11):1428-38. Epub 2014/09/30. doi: 10.1038/aps.2014.68. PubMed PMID: 25263338; PubMed Central PMCID: PMCPMC4220072.

38. Qie S, Ran Y, Lu X, Su W, Li W, Xi J, et al. Candesartan modulates microglia activation and polarization via NF-kappaB signaling pathway. *Int J Immunopathol Pharmacol* (2020) 34:2058738420974900. Epub 2020/11/26. doi: 10.1177/2058738420974900. PubMed PMID: 33237822; PubMed Central PMCID: PMCPMC7691946.

39. Torika N, Asraf K, Apte RN, Fleisher-Berkovich S. Candesartan ameliorates brain inflammation associated with Alzheimer's disease. *CNS Neurosci Ther* (2018) 24(3):231-42. Epub 2018/01/25. doi: 10.1111/cns.12802. PubMed PMID: 29365370; PubMed Central PMCID: PMCPMC6489976.

40. Xu Y, Xu Y, Wang Y, Wang Y, He L, Jiang Z, et al. Telmisartan prevention of LPS-induced microglia activation involves M2 microglia polarization via CaMKKβ-dependent AMPK activation. *Brain Behav Immun* (2015) 50:298-313. Epub 2015/07/19. doi: 10.1016/j.bbi.2015.07.015. PubMed PMID: 26188187.

41. Qiu Z, Lu P, Wang K, Zhao X, Li Q, Wen J, et al. Dexmedetomidine Inhibits Neuroinflammation by Altering Microglial M1/M2 Polarization Through MAPK/ERK Pathway. *Neurochem Res* (2020) 45(2):345-53. Epub 2019/12/12. doi: 10.1007/s11064-019-02922-1. PubMed PMID: 31823113.

42. Wu F, Luo T, Mei Y, Liu H, Dong J, Fang Y, et al. Simvastatin alters M1/M2 polarization of murine BV2 microglia via Notch signaling. *J Neuroimmunol* (2018) 316:56-64. Epub 2018/01/24. doi: 10.1016/j.jneuroim.2017.12.010. PubMed PMID: 29361314.

43. Wu SY, Xing F, Sharma S, Wu K, Tyagi A, Liu Y, et al. Nicotine promotes brain metastasis by polarizing microglia and suppressing innate immune function. *J Exp Med* (2020) 217(8). Epub 2020/06/05. doi: 10.1084/jem.20191131. PubMed PMID: 32496556; PubMed Central PMCID: PMCPMC7398164 outside the submitted work. Dr. Chan reported other from Elekta and other from Monteris outside the submitted work. No other disclosures were reported.

44. Cui W, Sun C, Ma Y, Wang S, Wang X, Zhang Y. Inhibition of TLR4 Induces M2 Microglial Polarization and Provides Neuroprotection via the NLRP3 Inflammasome in Alzheimer's Disease. *Front Neurosci* (2020) 14:444. Epub 2020/06/09. doi: 10.3389/fnins.2020.00444. PubMed PMID: 32508567; PubMed Central PMCID: PMCPMC7251077.

45. Pisanu A, Lecca D, Mulas G, Wardas J, Simbula G, Spiga S, et al. Dynamic changes in pro- and anti-inflammatory cytokines in microglia after PPAR-gamma agonist neuroprotective treatment in the MPTPp mouse model of progressive Parkinson's disease. *Neurobiol Dis* (2014) 71:280-91. Epub 2014/08/20. doi: 10.1016/j.nbd.2014.08.011. PubMed PMID: 25134730.

46. Li J, Dai X, Zhou L, Li X, Pan D. Edaravone Plays Protective Effects on LPS-Induced Microglia by Switching M1/M2 Phenotypes and Regulating NLRP3 Inflammasome Activation. *Front Pharmacol* (2021) 12:691773. Epub 2021/06/18. doi: 10.3389/fphar.2021.691773. PubMed PMID: 34135761; PubMed Central PMCID: PMCPMC8201503.

47. Apolloni S, Fabbrizio P, Parisi C, Amadio S, Volonte C. Clemastine Confers Neuroprotection and Induces an Anti-Inflammatory Phenotype in SOD1(G93A) Mouse Model of Amyotrophic Lateral Sclerosis. *Mol Neurobiol* (2016) 53(1):518-31. Epub 2014/12/09. doi: 10.1007/s12035-014-9019-8. PubMed PMID: 25482048.

48. Hu ZW, Zhou LQ, Yang S, Chen M, Yu HH, Tao R, et al. FTY720 Modulates Microglia Toward Anti-inflammatory Phenotype by Suppressing Autophagy via STAT1 Pathway. *Cell Mol Neurobiol* (2021) 41(2):353-64. Epub 2020/04/29. doi: 10.1007/s10571-020-00856-9. PubMed PMID: 32342246.

49. Choi SH, Aid S, Kim HW, Jackson SH, Bosetti F. Inhibition of NADPH oxidase promotes alternative and anti-inflammatory microglial activation during neuroinflammation. *J Neurochem* (2012) 120(2):292-301. Epub 2011/11/05. doi: 10.1111/j.1471-4159.2011.07572.x. PubMed PMID: 22050439; PubMed Central PMCID: PMCPMC3386526.

50. Liu X, Zhang X, Niu X, Zhang P, Wang Q, Xue X, et al. Mdivi-1 Modulates Macrophage/Microglial Polarization in Mice with EAE via the Inhibition of the TLR2/4-GSK3β-NF-κB Inflammatory Signaling Axis. *Mol Neurobiol* (2021). Epub 2021/10/08. doi: 10.1007/s12035-021-02552-1. PubMed PMID: 34618332.

51. Lin MS. CISD2 Attenuates Inflammation and Regulates Microglia Polarization in EOC Microglial Cells-As a Potential Therapeutic Target for Neurodegenerative Dementia. *Front Aging Neurosci* (2020) 12:260. Epub 2020/10/03. doi: 10.3389/fnagi.2020.00260. PubMed PMID: 33005144; PubMed Central PMCID: PMCPMC7479185.

52. Toscano R, Millan-Linares MC, Lemus-Conejo A, Claro C, Sanchez-Margalet V, Montserrat-de la Paz S. Postprandial triglyceride-rich lipoproteins promote M1/M2 microglia polarization in a fatty-acid-dependent manner. *J Nutr Biochem* (2020) 75:108248. Epub 2019/11/11. doi: 10.1016/j.jnutbio.2019.108248. PubMed PMID: 31707281.

53. Zhou Q, Lin L, Li H, Wang H, Jiang S, Huang P, et al. Melatonin Reduces Neuroinflammation and Improves Axonal Hypomyelination by Modulating M1/M2 Microglia Polarization via JAK2-STAT3-Telomerase Pathway in Postnatal Rats Exposed to Lipopolysaccharide. *Mol Neurobiol* (2021). Epub 2021/09/30. doi: 10.1007/s12035-021-02568-7. PubMed PMID: 34585328.

54. Ren C, Li D, Zhou Q, Hu X. Mitochondria-targeted TPP-MoS2 with dual enzyme activity provides efficient neuroprotection through M1/M2 microglial polarization in an Alzheimer's disease model. *Biomaterials* (2020) 232:119752. Epub 2020/01/11. doi: 10.1016/j.biomaterials.2019.119752. PubMed PMID: 31923845.

55. Xie L, Liu Y, Zhang N, Li C, Sandhu AF, Williams G, 3rd, et al. Electroacupuncture Improves M2 Microglia Polarization and Glia Anti-inflammation of Hippocampus in Alzheimer's Disease. *Front Neurosci* (2021) 15:689629. Epub 2021/10/15. doi: 10.3389/fnins.2021.689629. PubMed PMID: 34646113; PubMed Central PMCID: PMCPMC8502881.

56. Wang M, Feng LR, Li ZL, Ma KG, Chang KW, Chen XL, et al. Thymosin β4 reverses phenotypic polarization of glial cells and cognitive impairment via negative regulation of NF-κB signaling axis in APP/PS1 mice. *J Neuroinflammation* (2021) 18(1):146. Epub 2021/06/30. doi: 10.1186/s12974-021-02166-3. PubMed PMID: 34183019; PubMed Central PMCID: PMCPMC8240373.

57. Tang Y, Li T, Li J, Yang J, Liu H, Zhang XJ, et al. Jmjd3 is essential for the epigenetic modulation of microglia phenotypes in the immune pathogenesis of Parkinson's disease. *Cell Death Differ* (2014) 21(3):369-80. Epub 2013/11/12. doi: 10.1038/cdd.2013.159. PubMed PMID: 24212761; PubMed Central PMCID: PMCPMC3921590.

58. Yang X, Zhang Y, Chen Y, He X, Qian Y, Xu S, et al. LncRNA HOXA-AS2 regulates microglial polarization via recruitment of PRC2 and epigenetic modification of PGC-1α expression. *J Neuroinflammation* (2021) 18(1):197. Epub 2021/09/14. doi: 10.1186/s12974-021-02267-z. PubMed PMID: 34511122; PubMed Central PMCID: PMCPMC8436538.

59. Wasko NJ, Kulak MH, Paul D, Nicaise AM, Yeung ST, Nichols FC, et al. Systemic TLR2 tolerance enhances central nervous system remyelination. *J Neuroinflammation* (2019) 16(1):158. Epub 2019/07/29. doi: 10.1186/s12974-019-1540-2. PubMed PMID: 31351476; PubMed Central PMCID: PMCPMC6660683.

60. Lourbopoulos A, Grigoriadis N, Lagoudaki R, Touloumi O, Polyzoidou E, Mavromatis I, et al. Administration of 2-arachidonoylglycerol ameliorates both acute and chronic experimental autoimmune encephalomyelitis. *Brain Res* (2011) 1390:126-41. Epub 2011/03/17. doi: 10.1016/j.brainres.2011.03.020. PubMed PMID: 21406188.

61. Yu Z, Sun D, Feng J, Tan W, Fang X, Zhao M, et al. MSX3 Switches Microglia Polarization and Protects from Inflammation-Induced Demyelination. *J Neurosci* (2015) 35(16):6350-65. Epub 2015/04/24. doi: 10.1523/JNEUROSCI.2468-14.2015. PubMed PMID: 25904788; PubMed Central PMCID: PMCPMC6605213.

62. Aryanpour R, Zibara K, Pasbakhsh P, Jame'ei SB, Namjoo Z, Ghanbari A, et al. 17beta-Estradiol Reduces Demyelination in Cuprizone-fed Mice by Promoting M2 Microglia Polarity and Regulating NLRP3 Inflammasome. *Neuroscience* (2021) 463:116-27. Epub 2021/04/02. doi: 10.1016/j.neuroscience.2021.03.025. PubMed PMID: 33794337.

63. Li Z, Liu F, He X, Yang X, Shan F, Feng J. Exosomes derived from mesenchymal stem cells attenuate inflammation and demyelination of the central nervous system in EAE rats by regulating the polarization of microglia. *Int Immunopharmacol* (2019) 67:268-80. Epub 2018/12/21. doi: 10.1016/j.intimp.2018.12.001. PubMed PMID: 30572251.

64. Zhang J, Buller BA, Zhang ZG, Zhang Y, Lu M, Rosene DL, et al. Exosomes derived from bone marrow mesenchymal stromal cells promote remyelination and reduce neuroinflammation in the demyelinating central nervous system. *Exp Neurol* (2021):113895. Epub 2021/10/16. doi: 10.1016/j.expneurol.2021.113895. PubMed PMID: 34653510.
